# Sp1 Transcription Factor and GATA1 *cis*-Acting Elements Modulate Testis-Specific Expression of Mouse Cyclin A1

**DOI:** 10.1371/journal.pone.0047862

**Published:** 2012-10-24

**Authors:** Sunil K. Panigrahi, Ana Vasileva, Debra J. Wolgemuth

**Affiliations:** 1 Department of Genetics and Development, Columbia University Medical Center, New York, New York, United States of America; 2 Center for Radiological Research, Columbia University Medical Center, New York, New York, United States of America; 3 Department of Obstetrics and Gynecology, Columbia University Medical Center, New York, New York, United States of America; 4 Institute of Human Nutrition, Columbia University Medical Center, New York, New York, United States of America; 5 Herbert Irving Comprehensive Cancer Center, Columbia University Medical Center, New York, New York, United States of America; Università degli Studi di Milano, Italy

## Abstract

Cyclin A1 is a male germ cell-specific cell cycle regulator that is essential for spermatogenesis. It is unique among the cyclins by virtue of its highly restricted expression *in vivo*, being present in pachytene and diplotene spermatocytes and not in earlier or later stages of spermatogenesis. To begin to understand the molecular mechanisms responsible for this narrow window of expression of the mouse cyclin A1 (*Ccna1*) gene, we carried out a detailed analysis of its promoter. We defined a 170-bp region within the promoter and showed that it is involved in repression of *Ccna1* in cultured cells. Within this region we identified known *cis*-acting transcription factor binding sequences, including an Sp1-binding site and two GATA1-binding sites. Neither Sp1 nor GATA1 is expressed in pachytene spermatocytes and later stages of germ cell differentiation. Sp1 is readily detected at earlier stages of spermatogenesis. Site-directed mutagenesis demonstrated that neither factor alone was sufficient to significantly repress expression driven by the *Ccna1* promoter, while concurrent binding of Sp1, and most likely GATA1 and possibly additional factors was inhibitory. Occupancy of Sp1 on the *Ccna1* promoter and influence of GATA1-dependent *cis*-acting elements was confirmed by ChIP analysis in cell lines and most importantly, in spermatogonia. In contrast with many other testis-specific genes, the CpG island methylation status of the *Ccna1* promoter was similar among various tissues examined, irrespective of whether *Ccna1* was transcriptionally active, suggesting that this regulatory mechanism is not involved in the restricted expression of *Ccna1*.

## Introduction

The cyclins along with their cyclin-dependent kinase (CDK) partners form complexes that regulate eukaryotic cell cycle progression through the phosphorylation and activation of specific substrates [1]. It was discovered over ten years ago that there are two distinct A-type cyclins in the mouse (and human) genome which exhibit strikingly different patterns of expression [2]. Cyclin A2 (previously referred to as cyclin A) was originally identified in adenovirus transformed human cells as protein p60 and shown to be involved in cellular transformation through its association with E1A [3]. Cyclin A2 is ubiquitously expressed in mitotically dividing cells during embryogenesis and in a variety of adult tissues and is upregulated in a number of cancers [3], [4]. In contrast, the expression of cyclin A1 is highly restricted, being most abundant in the testis in both mice and human [2], [5], [6]. Cyclin A2 is responsible for regulation of both G1-S and G2-M transitions by binding to different CDK partners: CDK2 in S-phase and CDK1 during G2-phase of cell cycle, respectively [7], [8]. Cyclin A1 interacts with both CDK1 and CDK2 [9], and because of its limited distribution, can only be involved in the G2-M transition of meiosis I [10].

Both A-type cyclins are expressed in the testis; however their expression pattern is non-overlapping. Cyclin A2 is expressed in mitotically dividing cells within both the somatic [11] and germ cell lineages, in the latter case, spermatogonia [12]. In contrast, cyclin A1 is restricted to the germ line and specifically, to late pachytene and diplotene spermatocytes [2], [10], [13]. Loss of cyclin A1 function results in an arrest of spermatogenesis precisely at the end of meiotic prophase I [10], while cyclin A2 knock-out mouse embryos die shortly after implantation, consistent with its essential role in cell proliferation [14].

We recently showed that the requirement for cyclin A2 in cellular proliferation varies by cell type [15]. That is, mouse embryonic fibroblasts (MEFs) can proliferate in the absence of both A-type cyclins, with an apparent compensatory up-regulation of cyclin E. In contrast, cyclin A2 is essential for proliferation and differentiation in both hematopoietic stem cells and embryonic stem cells.

We had previously shown that in transgenic mice, a genomic fragment from −4800 bp to +800 bp of the mouse *Ccna1* gene consistently directs *lacZ* expression in male germ cells, preserving the temporal pattern of activation and repression in the appropriate differentiating cell types [16]. However, mice carrying a *Ccna1* promoter fragment comprising −1300 bp to +800 bp express *LacZ* in a similar pattern but in a less efficient manner, implying the presence of regulatory elements in this region. Moreover, the corresponding region of the human *CCNA1* promoter showed ectopic expression in spermatogonia as well as in sperm in transgenic mice [17]. Thus, although a region governing the expression of mouse *Ccna1* has been identified, the specific sequences, chromatin conformation, and associated transcription factors required for its unique activation and repression in male germ cells have not been studied.

To elucidate the transcriptional mechanisms underlying *Ccna1*’s repression, we analyzed its promoter region in cultured cells. We demonstrated that constructs carrying the full length promoter of *Ccna1* (−5000 bp to +330 bp) failed to drive reporter gene expression in both the mouse embryonic fibroblast-derived NIH3T3 cells and in the adult mouse testis-derived cell line, GC-4spc [18]. Sequential deletions revealed sustained repression when regions upstream of position −290 bp with respect to the transcription start site (TSS) were still present. A robust transcriptional activity was readily detected from a region encompassing −120 bp to +330 bp. Examination of the 170-bp fragment between −290 and −120 bp identified a single Sp1 binding site and two GATA1 binding sites, which mutational analysis revealed to be critical for the repression of *Ccna1* in these cell lines. The expression pattern of Sp1 and GATA1 proteins in the testis suggests that they may be involved in repressing *Ccna1* expression in the early germ cell lineage, and that their absence in spermatocytes may contribute to *Ccna1* activation in these cells.

## Materials and Methods

### Generation of Luciferase Reporter Constructs

The promoter fragment spanning −5000 bp to +330 bp of mouse *Ccna*1 was generated by PCR amplification from genomic DNA and cloned into pGL-Basic vector at *Kpn1* and *Bgl II* restriction sites. The start codon, ATG, was mutated to ATT to avoid initiation of translation. The remaining deletion constructs were generated by PCR amplification from the above plasmid and were introduced into an empty pGLbasic vector as above. Mutations of Sp1 and GATA1 binding sites in the deletion construct containing −440 bp to +330 bp of the *Ccna1* promoter were introduced by site directed mutagenesis (Stratagene) as per the manufacturer’s instructions. All deletions and point mutations were confirmed by DNA sequencing.

### Cell Culture and Transfections

NIH3T3 cells (CRL-1658™ ATCC, VA) and GC-4spc cells, provided by Dr Peter Burfeind [18] cells were cultured in Dulbecco’s modified Eagle’s medium supplemented with 10% fetal bovine serum containing 100 units/ml penicillin and 100 mg/ml streptomycin. For GC-4spc cells, 1x non-essential amino acids, 1 mM sodium pyruvate and 2 mM L-glutamine were added as supplements to the media. Transfection of plasmid DNA was carried out using Lipofectamine-2000 (LifeTechnologies, Inc.) into 24-well plates seeded with 1×10^5^ cells 24 hours prior to transfection. 1 µg of luciferase reporter plasmid was transfected together with 100 ng of a pRL-CMV vector used, to normalize for transfection efficiency. Cells were harvested 36 hours post-transfection and luciferase activity was measured by Dual luciferase assay (Promega) according to the manufacturer’s instruction. For siRNA transfection, both NIH3T3 and GC-4spc cells were plated in 12-well dishes and transfected with siRNA oligonucleotides specific for mouse Sp1 (sc-29488) and/or mouse GATA1 (sc-35452, Santa Cruz Biotechnology) at 50% confluency. Cells were incubated with serum and antibiotic-free media prior to transfection, while siRNA duplexes (100 µmole) were incubated with Lipofectamine 2000 transfection reagent (0.6% in OPTI-MEM I medium) at room temperature for 20 min. Complexes were then added to the cells and incubated for 6 hours at 37°C with 5% CO_2,_ followed by addition of serum and antibiotic to the medium. Cells were harvested 48 hours post-transfection, washed once with PBS and lysed in 100 µl of RIPA buffer (50 mM Tris-Cl pH7.5, 150 mM NaCl, 0.1% SDS, 0.5% Na deoxycholate, 1% NP-40 and protease inhibitors) and subjected to SDS–PAGE. For reporter gene experiments with siRNA, reporter constructs and siRNA were co-transfected into the cells. All experiments were carried out in duplicate and were performed independently at least thrice. Luciferase assay data are represented as the mean ± SD of three independent experiments.

### Cell Separation and Immunoblot

The protocol for separation of adult germ cells was adapted from our previous publication [19]_ENREF_9_ENREF_9. Briefly, adult germ cells (primary spermatocytes and haploid round spermatids) were isolated from mice at 4–6 weeks of age by enzymatic digestion. Decapsulated testes were minced into small pieces and digested with 1 mg/ml collagenase for 2 min at 37°C. The dispersed tubules were allowed to sediment, the supernatant was decanted, and the tubules were washed twice with 1× DPBS. The tubules were then incubated in 1× DPBS containing Trypsin (0.25 mg/ml) and DNase I (1 µg/ml) for 2 min at 37°C. The resulting cell suspension was gently sheared for 2 min with a Pasteur pipette and filtered through a 70 µm nylon mesh to remove cell aggregates. The cells were centrifuged at 4°C for 10 min at 200 g and the pellet was suspended in 20 ml of 1× DPBS containing 0.5% BSA. The cell suspension was adjusted to a final concentration of ∼5×10^6^ cells per ml. The cell suspension, fractionation, and pooling steps were all carried out at 4°C. The Celsep apparatus (Dupont Inc., Wilmington, Delaware) was loaded in the “up” (angled) position. The gradient was formed with 425 ml each of 2% and 4% BSA in 1×DPBS at a pump speed setting of ∼20 ml/min. The cell suspension was loaded with the chamber oriented in the “down” (horizontal) position and sedimentation was allowed to proceed for 95 min. The chamber was then reoriented to the “up” position and 10 ml fractions were collected at a pump speed of ∼15 ml/min. Aliquots were collected for flow cytometry analysis. Pools of primary spermatocytes or haploid round spermatids were obtained at a purity of more than 85%.

Proteins were extracted from cell lines, purified cell populations, and minced fragments of adult testes incubated for 15 min at 4°C with RIPA buffer (see above). After centrifugation at 15,000 × *g*, protein concentration in the clear supernatant was determined by the Bradford method. Tissue lysates (25µg/lane) were subjected to electrophoresis on SDS-polyacrylamide (PAGE) gels. Proteins were transferred to polyvinylidene fluoride (PDVF) membranes, blocked with 5% nonfat milk in TBST (0.9% NaCl, 0.1% Tween-20, 100 mM Tris-HCl, pH 7.5) and incubated with primary antibodies overnight at 4°C. After three washes with TBST, the membranes were incubated with appropriate secondary antibodies for 1 hour. After three additional washes with TBST, HRP activity was visualized with the Millipore Immobilon Western Blotting Detection kit per manufacturer’s instructions.

### Analysis of CpG Methylation

Methylation-Specific Polymerase Chain Reaction was used to assess total methylation levels in the *Ccna1* promoter in different mouse tissues using the Methyl-Profiler™ DNA Methylation PCR Array System (SABiosciences, Frederick, MD). DNA methylation-sensitive and methylation-dependent restriction enzymes were used to selectively digest unmethylated or methylated DNA, respectively. DNA remaining after digestion was quantified by real-time PCR using primers flanking the region of interest. The relative concentration of differentially methylated DNA (specifically hypermethylated, intermediately methylated, and unmethylated DNA) was determined by comparing the amount in each digest with that of a mock digest. The PCR cycling conditions were as follows: 1 cycle at 95°C for 10 min, 40 cycles including 15 seconds at 97°C and 1 min at 72°C. The PCR product was marked with SYBR Green. Data analysis was performed using the online data analysis system provided by the manufacturer.

Position-specific CpG methylation on the *Ccna1* promoter was analyzed by bisulfite conversion as described by Hajkova and colleagues [20]. For *Ccna1* promoter analysis, 1 µg of total genomic DNA was converted using a commercial kit (EZ DNA Methylation Gold™Kit; Zymo Research Corp., Orange, CA, USA). Fragments containing the *Ccna1* promoter CpG island were amplified from the converted DNA using primers spanning the CpG island. PCR fragments were gel purified and cloned into pGEMTeasy vector (Promega). For each sample five clones were sequenced and statistical comparison of bisulfite data was analyzed using QUMA software http://quma.cdb.riken.jp/ [21].

### Chromatin Immunoprecipitation (ChIP)

Chromatin immunoprecipitation was done as previously described [22] with minor modifications. Purified cell populations from testis and cell line samples were cross linked with 1% formaldehyde in PBS at 34°C for 20 min and the reaction was terminated by the addition of 125 mM glycine. DNA was sheared to 300–500 bp using sonication. Debris was removed by centrifugation at 16,000 × *g* for 10 min at 4°C and the supernatant was incubated with anti-Sp1 (Millipore, cat #07645), anti-RNA polymerase II (Millipore, cat# 05623) or anti-H3K27me3 (Abcam, cat# ab6002) antibodies and protein A/G beads at 4°C overnight. Mouse (for H3K27me3) or rabbit IgG (for Sp1 and RNA Polymerase II) was used in control experiments. The beads were washed and the chromatin was eluted and reverse cross-linked by incubation at 65°C. DNA was purified and used as a template for PCR detection.

### Electrophoretic Mobility Shift Assay (EMSA)

Nuclear extracts from mouse testis at postnatal day (pnd) 10, NIH3T3 and GC-4spc cells were prepared using a nuclear extract kit (Active motif) as per the manufacturer’s protocol. Nuclear extract (7.5 mg) was pre-incubated on ice for 5 min in the presence of binding buffer (15 mM HEPES, pH 7.9, 60 mM NaCl, 0.5 mM EDTA, 1 mM MgCl_2_, 2 mM DTT, 5% glycerol, and 0.2 mM PMSF), 1 µg poly dI-dC. NaCl present in the nuclear extracts was taken into account when calculating the final NaCl concentration. Gamma [^32^P]-ATP-labelled probe spanning -290/−120 bp of the *Ccna1* promoter (25,000 cpm) was added and the reactions were incubated on ice for an additional 30 min. Reactions were loaded onto 5% non-denaturing polyacrylamide gels that had been pre-run at 150 V for 45 min. Reactions were electrophoresed at 150 V and then blotted onto paper support, dried, and exposed to film at −80°C for 12–24 hours.

## Results

### The Region between −290 to −120 bp of the *Ccna1* Promoter is Responsible for Repressing Gene Expression in Cultured Cells

Our previous studies have clearly shown that transgenes carrying genomic fragments of mouse *Ccna1* spanning −4800 bp to +800 bp of the putative TSS were expressed specifically in spermatocytes at stages IX to XII, recapitulating the expression pattern of the endogenous mouse gene [16]. To map and identify regulatory elements within this region responsible for repression of *Ccna1* expression, we generated a series of deletion constructs spanning −5000 bp to +330 bp with respect to the TSS and monitored the activity of a luciferase reporter gene in two cultured cell models. The embryonic fibroblast-derived NIH3T3 cell line does not express cyclin A1 and was selected to reflect generic repression in a somatic cell lineage. The GC-4spc cell line was derived from germ cells of adult mouse testes and expresses several genes characteristic of meiotic prophase, including Pgk2, proacrosin, and A-myb [18]. However, northern blot hybridization and immunoblot analyses revealed that cyclin A1 is not expressed (Figure S1), again representing a useful model for identifying repressive elements.

**Figure 1:**
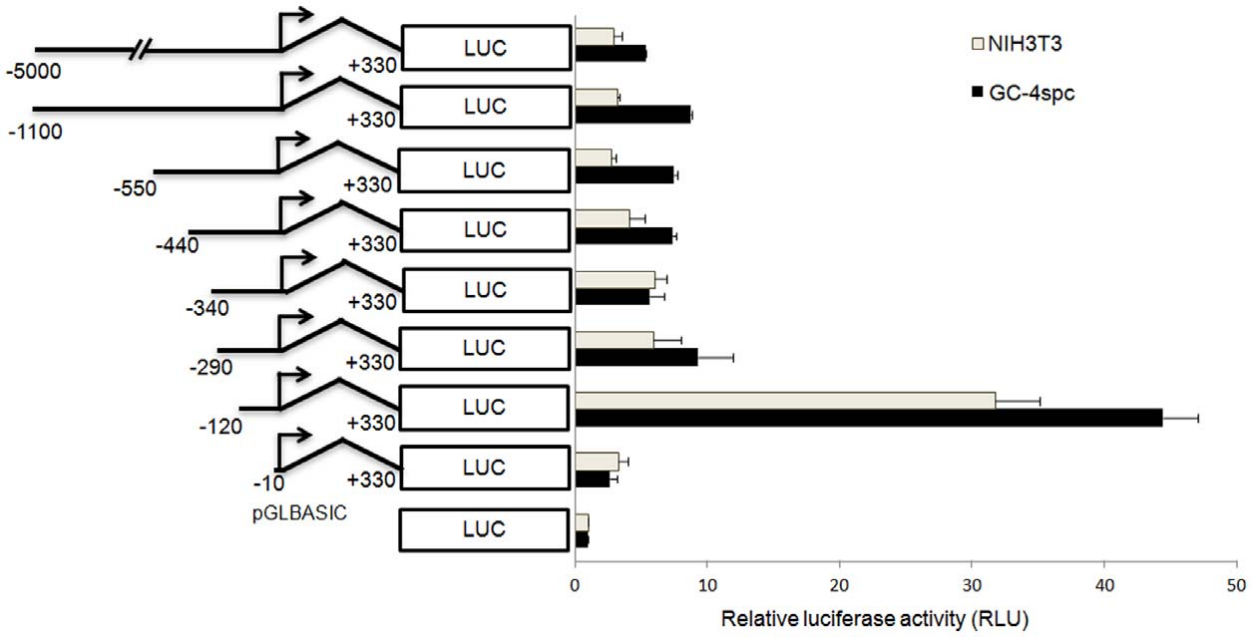
5′ deletion analysis of the *Ccna1* promoter reveals a repressor element upstream of -120 bp with respect to the transcription start site (TSS). Chimeric constructs of the *Ccna1* promoter-luciferase reporter are schematically represented on the category-axis. The upstream and downstream endpoints of each fragment are indicated with respect to the TSS, which is indicated by a bent arrow. Constructs were tested for transcriptional activity in GC-4spc and NIH3T3 cells as described in the Methods section. The luciferase activity obtained for the pGL-Basic vector, in which a functional promoter was absent, was set at 1.0 and all other luciferase activities were expressed as fold of the values obtained for pGL-Basic. All experiments were performed in quadruplicate, and the means ± standard deviations of luciferase expression from independent experiments are shown. In all experiments the luciferase activity of pRL-CMV, *Renilla* luciferase expressing under CMV promoter was used as an internal control.

As assessed 36 hours post-transfection using a dual luciferase assay, constructs carrying sequentially shorter fragments with 5′ ends from −5000 bp to −290 bp showed either essentially a basal (NIH3T3 cells) or very low (GC-4spc cells) levels of luciferase expression (Figure 1). However, an ∼6-fold increase in luciferase activity was observed in both cell lines with the construct spanning −120 to +330 of the *Ccna1* promoter, compared to the construct containing the −290 bp to +330 bp fragment (Figure 1). Not unexpectedly, the shortest -10/+330 fragment lacked identifiable promoter function as luciferase activity fell to or slightly below basal levels. Therefore, the region between −290 bp to −120 bp of the *Ccna1* promoter contains important *cis*-acting repressor elements that suppress *Ccna1* expression in GC-4spc and NIH3T3 cells.

### Identification of Putative Transcription Factor Binding Sites in the −290/−120 bp Region of the *Ccna1* Promoter

**Figure 2:**
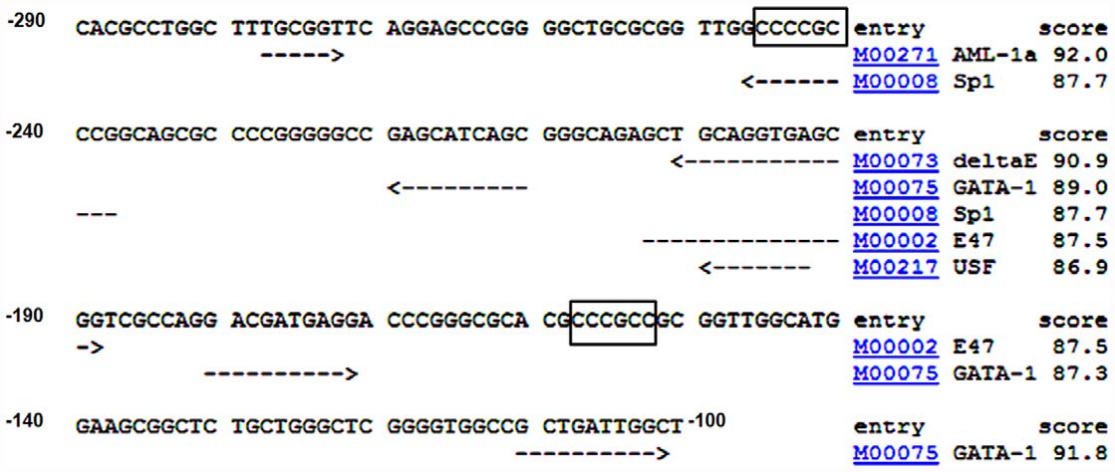
Identification of transcription factor consensus sequences in the *Ccna1* promoter. The −290/−100 region was screened for binding sites using TRANSFAC, a mammalian transcription factor databas, using a cut-off score of 85. Consensus sequences are underlined with dashed arrows and GC boxes are enclosed within rectangles.

Given our finding that the −290 bp to −120 bp (−290/−120) region is key to the transcriptional repression of *Ccna1,* we sought to identify transcription factors that could be involved in this regulation. Using the TRANSFAC database, we identified several putative transcription factor binding sequences in the −290/−100 region, including sites for AML-1a, Sp1, delta E, USF, E47 and GATA1 (Figure 2). Of these putative regulators, E47 (the E-box binding protein) and USFs (upstream stimulatory factors) are expressed in the testis but primarily in Sertoli cells. E47 regulates the expression of transferrin, a well known Sertoli cell differentiation marker [23], while USFs regulate *Nr5a1/Sf1* and *Shbg*, which are also turned on in differentiating Sertoli cells [24]. AML-1a is highly expressed in multiple leukemia cell lines [25] but its expression in male germ cells is not known. Delta E, also known as the YY1 transcription factor, is ubiquitously expressed, and in the testis is expressed in spermatogonia, spermatocytes, Sertoli cells and Leydig cells [26]. Sp1, the Sp family transcription factor binds to GC-rich sequences including GC-boxes 5′-(G/T)GGGCGG(G/A)(G/A)(C/T)-3′ and plays a major role in cell growth and tumor progression by regulating many cell cycle specific genes [27]. GATA1, a member of GATA family transcription factors, is required for erythroid and megakaryocytic differentiation [28], [29], and is best known for epigenetic transcriptional repression rather than transcriptional activation [30]. In testis, GATA1 expression has been reported in Sertoli and Leydig cells [31], [32] but not spermatocytes and spermatids [31]. Importantly, neither GATA1 nor Sp1 are expressed in pachytene spermatocytes [31], [33], which strongly express *Ccna1*, while both factors are readily detected in somatic cells and in lysates of juvenile testes. Sp1 is also expressed in spermatogonia and preleptotene spermatocytes [33]. The expression pattern of Sp1 and GATA1 lead us to hypothesize that these two factors might contribute to the repression of *Ccna1* in somatic cells and pre-meiotic germ cells and therefore selected them for further study.

As a first step in this analysis, we confirmed the reported expression of these two factors in NIH3T3 cells and in liver and determined their expression in GC-4spc cells, purified pachytene spermatocytes, purified round spermatids, and different stages of post-natal developing testes by immunoblot analysis (Figure S2). As expected, both factors are expressed in NIH3T3 cells and in liver. Furthermore, both are expressed in GC-4spc cells and in pnd10 and pnd18 testes. Neither factor is expressed in purified primary spermatocytes and round spermatids. By pnd28 and pnd45 the relative proportion of these cells increases substantially, resulting in undetectable levels of the proteins in testes of these ages. Comparison of their expression with that of cyclin A1 revealed the reciprocal nature of expression: Sp1 and/or GATA1 are expressed when cyclin A1 is not present and vice versa. This inverse correlation was not seen, however, in the post-meiotic stages of spermatogenesis, suggesting that another mechanism of *Ccna1* repression must be functioning. Detection of actin and Brdt served as positive controls for integrity of the proteins in immunoblot samples.

### Testis and a Germ Cell Line Contain Factors Binding to Sp1 and GATA1 Motifs in the *Ccna1* Promoter

**Figure 3:**
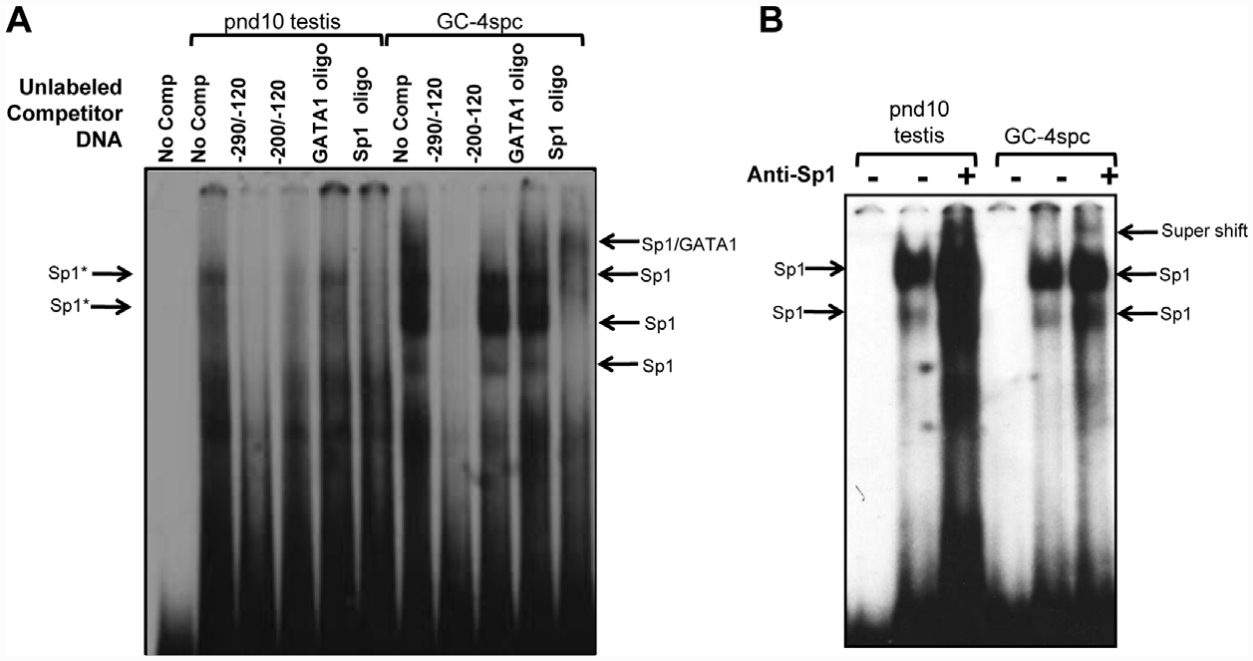
The minimum region of the *Ccna1* promoter necessary for protein binding resides within positions −**290/−120.** (A) EMSA were performed in nuclear extracts (NE) from pnd10 testis and NIH3T3 and GC-4spc cells incubated with a DNA probe of the −290/−120 bp region of the of *Ccna1* promoter. “No comp” designates lanes without cold competitor. For competition assays lysates were incubated with 100-fold excess of the indicated unlabelled DNA prior to addition of the labeled −290/−120 probe. Formation of DNA-protein complexes in reactions with labeled −290/−120 probe alone was abolished completely by the addition of the same unlabeled competitor (lanes −290/−120). Oligo spanning the −200/−120bp region, ds oligo containing GATA1 binding site or an oligo containing Sp1 binding site did not or only partially compete with the −290/−120 probe for binding to transcription factors. Arrows indicate protein-DNA complexes that lead to the electrophoretic mobility shift. (B) DNA probes from the *Ccna 1* promoter (positions −290 to −120) were incubated with nuclear extract (NE) from pnd10 testis and GC-4spc cells. Addition of anti-Sp1 antibody is indicated with a “+”. Arrows point to supershift products of the Sp1-DNA probe complex bound to the antibody.

To elucidate the functional significance of the Sp1 and GATA1 binding sites within the -290/−120 region of the *Ccna1* promoter we carried out a competitive electrophoretic mobility shift assay (EMSA) with nuclear extracts from pnd10 testis and GC-4spc cells (Figure 3). Using a labeled oligonucleotide fragment corresponding to −290/−120 bp of the *Ccna1* promoter, a shift was observed in both testicular and GC-4spc lysates, indicating the formation of DNA-protein complexes (Figure 3A, lane labeled “No comp”). In the pnd10 extracts, the DNA-protein complexes were competitively excluded by pre-incubation of the lysate with 100-fold excess of the unlabeled probe (Figure 3A, lane −290/−120), while a slightly shorter competing oligo (−200/−120), containing two GATA1 binding sites but lacking the Sp1 site was less effective in the competition assay. This shorter DNA competed out only the larger complexes (indicated as Sp1*) but not the smaller ones (indicated as Sp1), which may lack GATA1 or other factors. These data suggest that Sp1 and GATA1 or other transcription factors bind concomitantly on the *Ccna1* promoter.

In GC-4spc lysates, a similar but not identical formation of complexes was observed (Figure 3A, GC-4spc, lane “No comp”). All protein–DNA complexes were competed by the −290/−120 oligo (lane −290/−120), but again, less effectively with the shorter oligo. The size difference of the shifted products most probably reflects differences in the composition of these complexes in pnd10 testis and GC-4spc cells. Notably, competition with either Sp1-specific or GATA1-specific oligos individually was ineffective in disrupting the complexes in testicular lysates (Figure 3A, pnd10 testis, lanes GATA1 oligo and Sp1 oligo), whereas in GC-4spc extracts the Sp1 oligo partially competed out the shifted bands while GATA1 alone was less effective (Figure 3A, GC-4spc, lanes GATA1 oligo and Sp1 oligo). The ineffective competition by GATA1 oligos suggests that other proteins/transcription factors may bind preferentially to the -200/−120 oligo. Once again, these data support the notion that the concomitant presence of more than one transcription factor on the *Ccna1* promoter is necessary for its full repression. Further, a supershift was observed when the EMSA reactions were incubated with antibodies to Sp1 (Figure 3B). Antibodies suitable for analysis of GATA1 binding were not available.

**Figure 4:**
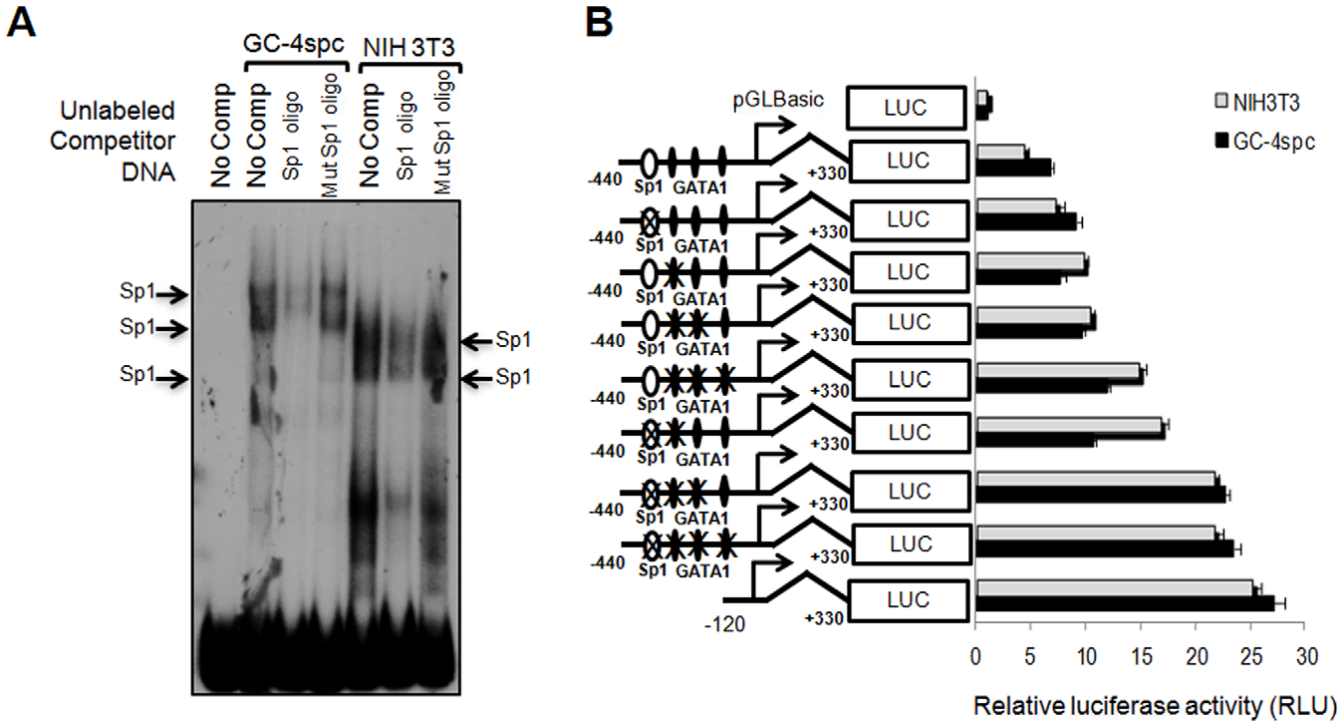
Sp1 and possibly GATA1 bind concomitantly to the *Ccna1* promoter and suppress its activity *in vivo*. (A) EMSA assay in nuclear extracts from indicated cell lines using radioactively labeled probe −290/−120 bp of the *Ccna 1* promoter shows specific DNA-protein complex formation (arrows). Complex formation was significantly reduced by the addition of 100-fold molar excess of unlabeled DNA competitor containing an intact Sp1 site while cold competitor containing mutant Sp1 recognition site failed to disrupt the binding. (B) Firefly luciferase reporter assay reveals binding to both Sp1 and GATA1 sites suppresses activation of the *Ccna1* promoter (constructs shown schematically to the left). Sp1 and GATA1 binding sites were marked as an open ellipse and black diamond, respectively. Crosses (“X”) indicate mutated binding sites. As an internal control, pRL-CMV expressing *Renilla* luciferase under the CMV promoter was co-transfected in each assay. Bars show the average luciferase activity of three independent experiments, normalized with respect to the *Renilla* luciferase activity.

To ensure that the complexes in the above experiments were formed specifically by Sp1 binding to its putative site on the *Ccna1* promoter, we performed additional EMSA using labeled 290/−120 oligonucleotides and unlabeled competitors containing mutated or intact Sp1 consensus sites in nuclear extracts from NIH3T3 and GC-4spc cells (Figure 4A). Shifted bands, which were detected in GC-4spc and NIH3T3 nuclear extracts using the −290/−120 probe (Figure 4A, lanes labeled “no comp”) were significantly reduced following addition of wild type Sp1 competitor (lanes labeled “Sp1 oligo”) but not with mutant Sp1 oligos (lanes “mut Sp1 oligo”). Since an oligo containing a single GATA1 site did not compete effectively in either testis or GC-4spc nuclear extracts, it was excluded from this competition experiment.

### Factors Binding to GATA1 Sites Contribute to Effective Sp1-mediated Repression of *Ccna1*

To test if the binding of Sp1 to the *Ccna1* promoter correlates with repression of reporter gene expression, we incorporated point mutations into the Sp1 binding site of a *Ccna1* promoter construct containing base pairs −440 to +330. Mutation of the Sp1 site alone failed to produce a significant elevation in luciferase expression (Figure 4B), which suggests that by itself, Sp1 cannot suppress *Ccna1* efficiently. To assess whether Sp1 and GATA1 sites together are required for repression of *Ccna1*, each of the binding sites within the −440/+330 bp region of the *Ccna1* promoter was mutated individually or in combinations (Figure 4B). Reporter constructs containing mutations either of the Sp1 binding site alone or one or two of the GATA1 binding sites did not show a significant change in luciferase activity as compared to the wild type −440/+330 bp construct (Figure 4B). However, when at least two GATA1 and the Sp1 binding sites were mutated, luciferase expression increased to levels similar to that produced by the −120 to +330 bp construct, which lacks these binding sites (Figure 4B). An intermediate level of luciferase expression was observed when all three GATA1 sites but not the Sp1 site were mutated or when a single GATA 1 site and the Sp1 site were both mutated.

**Figure 5:**
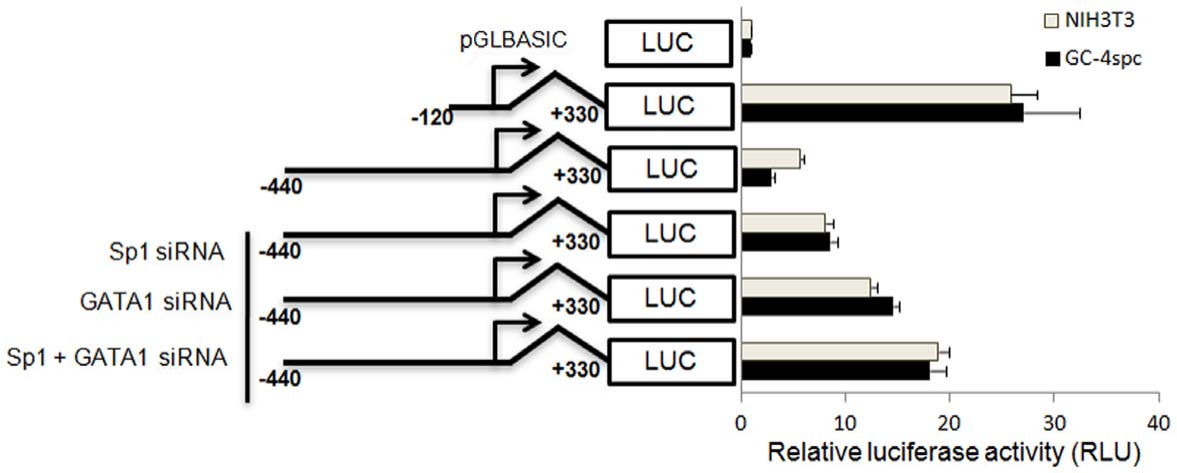
Suppression of SP1 and GATA1 expression leads to up-regulation of *Ccna1* promoter activity. The reporter constructs used for transfection are shown schematically on the left. Sp1 and GATA1 binding sites are marked with white ellipses and black diamonds, respectively. Reporter plasmids and corresponding siRNA oligonucleotides (100 µM) were co-transfected into NIH3T3 and GC-4spc cells. In all experiments pRL-CMV was co-transfected as an internal control. Bars show the average luciferase activity of five independent experiments, normalized with respect to the *Renilla* luciferase activity.

To further confirm the additive effect of Sp1 and GATA1 binding to sites on *Ccna1* regulation, the wild type −440/+330 bp construct was co-transfected with siRNA against Sp1 and GATA1 (Figure 5). Both the siRNAs, effectively and specifically downregulate the expression of their target genes, i.e. Sp1 and GATA1, in the cell lines (Figure S3). Addition of siRNA against each transcription factor resulted in a maximum of 50% de-repression (Figure 5). Evidence for the additive effect of Sp1 and GATA1 binding on *Ccna1* repression was observed upon administration of both siRNAs into the cells, which resulted in 70% de-repression of reporter gene expression. These results suggest that, either Sp1 alone or factor(s) binding to GATA1 consensus sequences are sufficient for partial repression of *Ccna1* expression, whereas co-occupancy of Sp1 and GATA1 sites results in efficient repression of cyclin A1 expression.

### Sp1 and Possibly GATA1 Bind to the *Ccna1* Promoter *in vivo*

**Figure 6:**
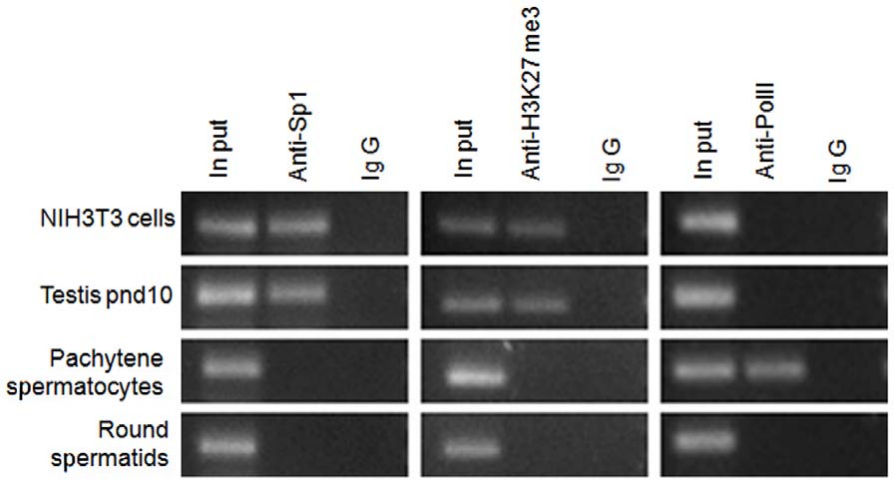
Identification of protein complexes by ChIP analysis demonstrates cell-specific binding of Sp1 and active RNA polymerase II to the *Ccna1* promoter as well as an enrichment of H3K27me3. Antibodies to Sp1, H3K27me3 or the corresponding IgG control were used to immunoprecipitate protein/DNA complexes from sonicated lysates of indicated cells or a tissue equivalent to 10^6^ cells in each case. After reversing the cross-linking, bound DNA was isolated and PCR was performed using primers that amplify the −290 to -100 bp region of the *Ccna1* promoter to detect bound Sp1 and H3K27me3 and the –120 to +90 bp region of the *Ccna1* promoter to detect RNA polymerase II binding.

To determine whether Sp1 and GATA1 bind to the *Ccna1* promoter *in vivo*, we performed ChIP assays on chromatin obtained from testicular cells, including extracts from pnd 10 mice (enriched in spermatogonia but also containing somatic cell types), purified pachytene spermatocytes, and purified round spermatids, utilizing reportedly ChIP grade antibodies for Sp1 [34] and GATA1 [35]. Unfortunately, the GATA1 antibodies did not yield reliable data in our hands. However, since GATA1 is known to be associated with the polycomb repressive complex 2 (PRC2) and H3K27me3, which are involved in silencing of various non-erythroid genes during differentiation [30], we performed ChIP assays with anti-H3K27me3 instead, to reflect the possible occupancy of GATA1 on the *Ccna1* promoter. Chromatin from the various samples was incubated with antibodies to Sp1 and H3K27me3 and the -290/+100 region was amplified by PCR. We observed binding of Sp1 and elevated levels of H3K27me3 in both NIH3T3 cells and pnd10 testis (Figure 6, top two rows in the left and middle panels, respectively). Consistent with the pattern of *Ccna1* expression, Sp1 binding or H3K27me3 enrichment was absent from the promoter in pachytene spermatocytes (third row, left and middle panels). As predicted, due to lack of GATA1 and Sp1 expression in round spermatids (Figure S2), binding to the *Ccna1* promoter was not observed with either Sp1 or H3K27me3 antibody (bottom row, left and middle panels), similar to the control IgG lanes (IgG lanes in the right panel). These data strongly suggest that Sp1-bound and H3K27me3 enriched (perhaps indicating GATA1 binding) chromatin is present at the *Ccna1* promoter of juvenile testis and in cell lines of somatic origin, but not in meiotic and post-meiotic cells. Conversely, a ChIP assay with RNApol II antibody as a marker for active transcription assembly amplified *Ccna1* products only from pachytene cell chromatin, where *Ccna1* is known to be expressed (Figure 6, far right panels). Therefore enrichment of Sp1 and H3K27me3 correlates inversely with the expression of the *Ccna1* gene, in a cell-specific manner.

### Tissue Specific *Ccna1* Expression is Not Regulated by Differential CpG Island Methylation

CpG island containing promoters can be classified into three categories: high CpG promoter (HCP), intermediate CpG promoter (ICP) and low CpG promoter (LCP) [36]. Tissue-specific gene expression of somatically expressed genes tends to be modulated by differential methylation at ICP promoters and usually does not correlate with the methylation status of HCP and LCP promoters. There are several examples with regard to male germ cell-specific genes wherein their expression is regulated by CpG island methylation on the promoter [37]–[40]. In somatic cells almost all CpG islands on the promoters of testis-specific genes are hypermethylated irrespective of the density of their CpG islands [38], [41]. Since most of the germ line-specific genes fall into the HCP class [36], the correlation between CpG island density and gene expression observed in somatic cells does not apply to the germ line.

**Figure 7:**
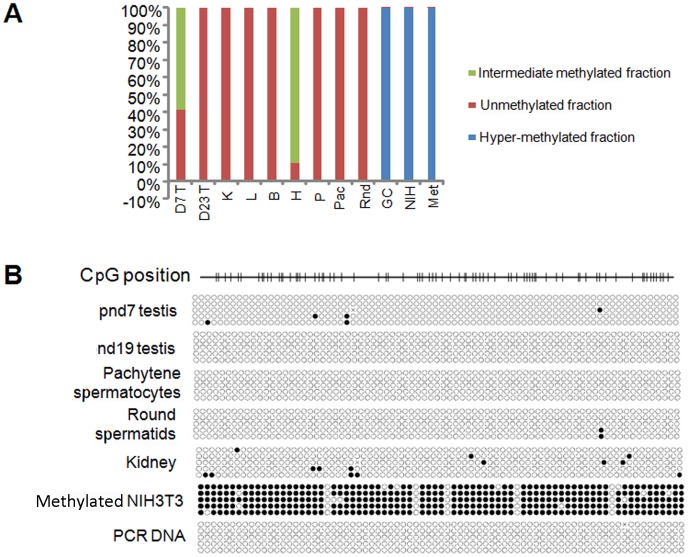
Tissue-specific *Ccna1* expression is not modulated by differential promoter methylation. (A) Gross methylation analysis of the *Ccna1* promoter in tissues and cell lines as follows: mouse testis (T) at the indicated age, kidney (K), liver (L), brain (B), heart (H), pancreas (P), purified pachytene cells (Pac), round spermatids (Rnd) and cell lines GC-4spc (GC), NIH3T3 (NIH). For each analysis, 500 ng of total genomic DNA was divided into four and assigned to mock control, methylation-sensitive, methylation-dependent and double digestion respectively. After digestion with methylation sensitive and insensitive nucleases, the methylation profile was obtained from the Ct values of real-time PCR with *Ccna1*-CpG methylation specific primers. (B) The methylation status of individual CpG residues on the *Ccna1* promoter was analyzed by bisulfite sequencing. One µg of DNA from the indicated samples was subjected to bisulfite conversion followed by amplification with primers specific for the *Ccna1* CpG island. PCR products were cloned into a pGEMTeasy vector, sequenced, and sequences were analyzed with QUMA software. *In vitro* CpG methylated NIH3T3 DNA was used as a positive control and a PCR amplified DNA from unconverted sequence was used as a negative control. Non-methylated CpG are indicated as open circles. The *Ccna1* promoter with CpG positions is depicted schematically.

As the *Ccna1* promoter also belongs to the HCP class and *Ccna1* is expressed only in testis, we thus hypothesized that in addition to repression by specific transcription factors, CpG methylation might contribute to the restricted expression of mouse *Ccna1*. We initially determined overall differential levels of methylation in the mouse *Ccna1* promoter region by a methyl profiler PCR assay [42]. Levels of methylation did not correlate with *Ccna1* expression in any of the tissue or testicular cell samples we tested, including purified pachytene spermatocytes and round spermatids, where the *Ccna1* promoter was either hypo-methylated or intermediately-methylated, or in the two cell lines (NIH3T3 and GC-4spc), where it was hyper-methylated (Figure 7A). However, since this method determines only the overall methylation status of a portion of the CpG island region of the promoter rather than the whole CpG island, we were unable to determine the methylation state of each CpG position.

Therefore, we subsequently performed bisulfite conversion and sequencing to obtain a detailed picture of the methylation status of the *Ccna1* promoter in various tissues, purified cell populations and cell lines (Figure 7B). In all tissue samples and the purified pachytene spermatocytes and round spermatids, most or all of the CpG positions were unmethylated. *In vitro* methylated NIH3T3 DNA served as a positive control. These results strongly suggest that CpG methylation does not correlate with tissue-specific *Ccna1* expression.

## Discussion

Cyclin A1 is essential for spermatogenesis and is expressed in a remarkably restricted pattern, being present at high levels uniquely in pachytene and diplotene spermatocytes. The *cis*-regulatory elements required for this highly restricted expression of the *Ccna1* gene have not been identified, but will likely involve both activation and repression. To identify these sequences we used serial deletions of *Ccna1* sequences upstream of the promoter, spanning -290/−120 bp and defined one such functional region that appears to contain elements necessary for its repression in somatic cells and most likely in early stages of spermatogenesis. Expression of reporter constructs containing part of the *Ccna1* promoter were critically dependant on one Sp1 and two GATA1 binding sites within this region. Specifically, our transfection analysis revealed a de-repression of *Ccna1* when both Sp1 and GATA1 binding sites were mutated. By gel-shift assays, we confirmed that Sp1 binds to the repressive region of the *Ccna1* promoter and that oligos containing a mutated Sp1 consensus site did not disrupt the binding. Importantly, we have demonstrated the actual *in vivo* association of Sp1 and H3K27me3 (a histone marker associated with GATA1 repression) with the *Ccna1* promoter in pnd 10 testis and cell lines and lack of such binding in pachytene spermatocytes by ChIP assays. This interaction correlates inversely with the transcriptional activity of *Ccna1 in* *vivo,* suggesting that Sp1, and possibly GATA1 or other factors indeed mediate the down regulation of cyclin A1 in somatic cells.

Our previous studies showed that the *Ccna1* promoter lacks a TATA box [16]. TATA-less promoters depend on GC-boxes for their trans-activation by interacting with Sp family member proteins [43] but repression via Sp factor binding to GC-boxes has not been reported. We predicted two putative GC-boxes in the −290/−120 bp region of the *Ccna1* promoter, one of which lies within the Sp1 binding site (Figure 2). Sp1 is highly expressed in spermatogonia and preleptotene spermatocytes, but its expression sharply drops in early pachytene [33], just prior to the onset of cyclin A1 expression. This inverse correlation between Sp1 and cyclin A1 expression has also been found for several other meiosis-specific genes, all of which have Sp1 binding sites and/or GC boxes in their proximal promoters and which are also activated at highest levels in pachytene spermatocytes. These genes include *H1t* [44], *Hspa2* [45], *Pgk2* [46], *Pdha2* [47], and *Ldh2* [48]. It has been suggested [33] that expression of these genes might be activated by Sp1/Sp3 factors, which is curious as Sp1 is clearly not present when they at the peak of expression. It should also be recalled that none of these studies actually assessed this putative activation directly, *in vivo*. Such activation of transcription is in stark contrast with the regulation of *Ccna1*, which does not appear to be transcribed prior to the late pachytene stage of spermatogenesis [2]. Furthermore, our *in vivo* ChIP data clearly demonstrate the absence of Sp1 on the *Ccna1* promoter in pachytene spermatocytes.

Methylation of DNA is known to be associated with the regulation of tissue-specific gene expression [49], [50]. In somatic cells tissue-specific gene expression is regulated by differential CpG methylation only on promoters classified as ICP and not HCP or LCP, a rule which does not apply to germline-specific genes [36]. For example, the promoters of SF1 [51], Ant 4 [52] and *PDHA2* [37] fall into the HCP class whereas *Tex 12*, tubulin 3, tubulin 7 [38] and *H1t* [53] fall into the LCP class and the expression of all these genes is repressed in somatic cells through CpG methylation of their promoters. Expression of the mouse *Tact1* gene is repressed in somatic cells by methylation of CpG dinucleotides in its open reading frame [54]. The human testis-specific gene lactate dehydrogenase c (*LDHc*), which is expressed only in germ cells, is regulated by transcription factors Sp1, CREB and also by CpG methylation [55]. The methylation of CpG dinucleotides has been shown to induce transcriptional repression by recruiting histone deacetylases, a process mediated by proteins of the methylated CpG binding domain (MBD) family [56].

In human testis, cyclin A1 is expressed in the late meiotic prophase, a pattern similar to its expression in mice [57]. Studies of the human *CCNA1* promoter reveal that a short fragment (from −190 bp to +145 bp) exhibits high transcriptional activity in HeLa cells [58] and this transcriptional activity is associated with binding of SP family transcription factors to the GC-boxes within that region. It was reported that the CpG methylation on the human *CCNA1* promoter does not correlate with its tissue-specific expression [59]. On the other hand, it has also been reported that up-regulation of *CCNA1* in cervical cancer is due to demethylation of the CpG island in its promoter [60], [61]. This finding suggests the possibility of epigenetic regulation of the tissue-specific expression of mouse cyclin A1. Our results, however, show no correlation between *Ccna1* expression and the extent of methylation of specific CpGs or CpG island methylation of the promoter in all the tissues and cell lines we tested. This finding is in agreement with previously reported data on the regulation of the human *CCNA1* gene, which suggest that unlike in cancer cells, CpG island methylation has no role in tissue-specific expression of cyclin A1 in normal tissues [59].

It will also be interesting in the future to study the regulatory mechanism involved in the downregulation of cyclin A1 in post-diplotene stages of spermatogenesis where neither Sp1 nor GATA1 are expressed. One obvious candidate is Sp3 which is expressed in round spermatids [33]. Sp3, the other Sp family member, binds to the same DNA sequence as Sp1 [62] and is known to be involved in gene repression by recruiting histone deacetylases (HDAC1, HDAC3 or HDAC4) to promoters [63]–[65]. In a preliminary study, we observed binding of HDAC1 to the Sp1 binding site of *Ccna1* promoter in round spermatids, suggesting the above mechanism might indeed contribute to the downregulation of *Ccna1* at post-diplotene stages of spermatogenesis. Further studies will be needed to understand the activator proteins required for its robust expression in the late-pachytene stage.

## Supporting Information

Figure S1 **Cyclin A1 is not expressed in GC-4spc cells.** (A) Northern analysis of total RNA isolated from GC-4spc (G), adult testis (T), heart (H), liver (L) with a *Ccna1* specific probe. Ethidium bromide stained 18s rRNA was used as loading control. (B) Immunoblot of total lysate (25 µg per lane) from GC-4spc (G), adult testis (T), heart (H), liver (L) and NIH3T3 (N) cells with antibodies to Cyclin A1. Actin was used as a loading control.
(TIF)

Figure S2 **SP1 and GATA1 are expressed in mitotic, but not meiotic and post-meiotic cells.** Immunoblots of total lysates (25 µg per lane) from testis pnd10, pnd18, pnd28, pnd45, liver, purified pachytene spermatocytes (PS-cytes), round spermatids (RS-tids), NIH3T3 and GC-4spc cells with antibodies to SP1, GATA1, *Ccna1* and *Brdt*. Actin was used as loading control. Both pachytene spermatocytes and round spermatids express an actin variant, undetectable by this antibody.
(TIF)

Figure S3 **Downregulation of Sp1 and GATA1 expression.** Immunoblot analysis verified the downregulation of Sp1 and GATA1 expression upon siRNA transfection.
(TIF)
