# Supplementary figures and images for: Sp1 Transcription Factor and GATA1 cis-Acting Elements Modulate Testis-Specific Expression of Mouse Cyclin A1
Source: PLoS One. 2012 Oct 24;7(10):e47862. doi: 10.1371/journal.pone.0047862 (PMC3480434; doi:10.1371/journal.pone.0047862)

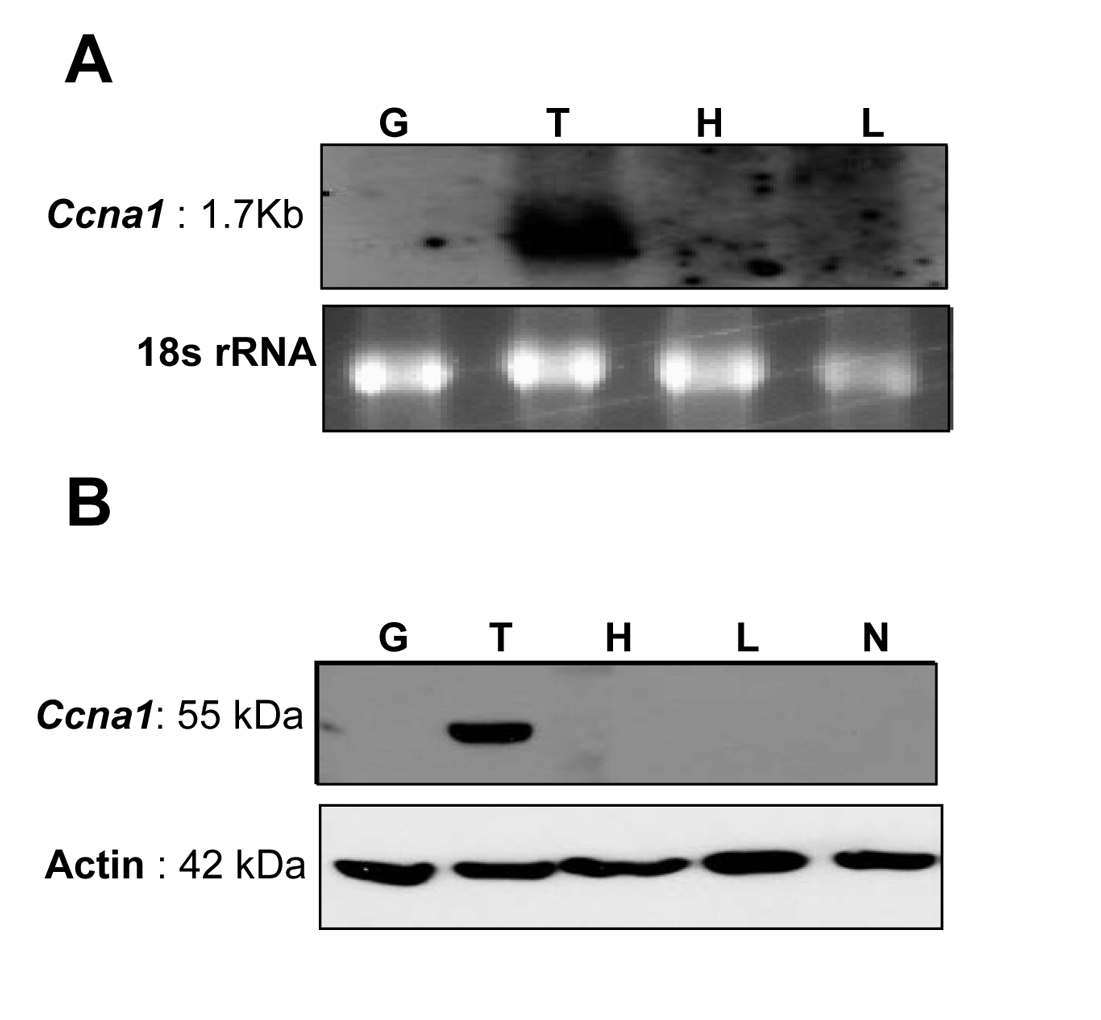

Supplement: Figure S1 — Cyclin A1 is not expressed in GC-4spc cells. (A) Northern analysis of total RNA isolated from GC-4spc (G), adult testis (T), heart (H), liver (L) with a Ccna1 specific probe. Ethidium bromide stained 18s rRNA was used as loading control. (B) Immunoblot of total lysate (25 µg per lane) from GC-4spc (G), adult testis (T), heart (H), liver (L) and NIH3T3 (N) cells with antibodies to Cyclin A1. Actin was used as a loading control. (TIF) [file pone.0047862.s001.tif]

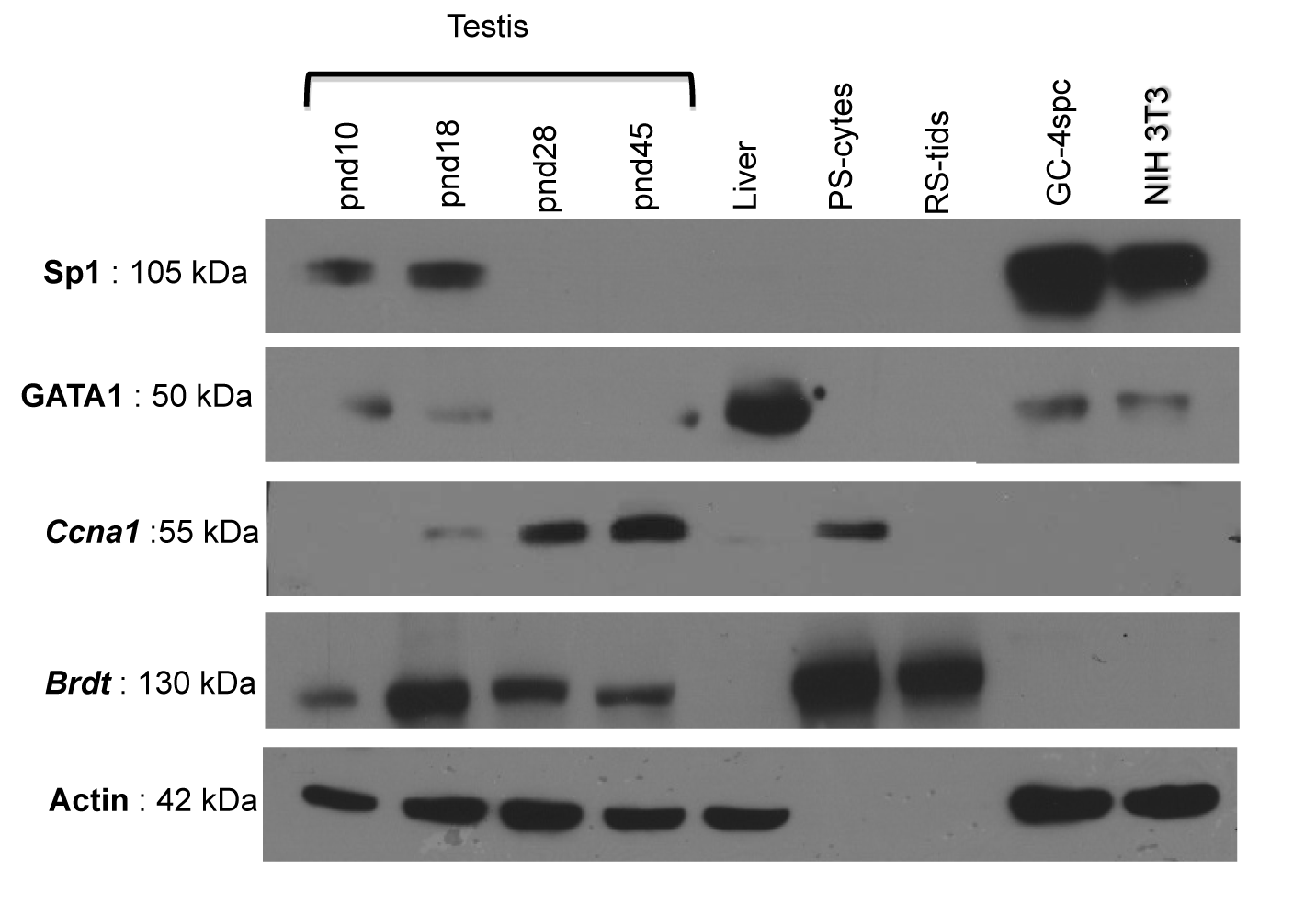

Supplement: Figure S2 — SP1 and GATA1 are expressed in mitotic, but not meiotic and post-meiotic cells. Immunoblots of total lysates (25 µg per lane) from testis pnd10, pnd18, pnd28, pnd45, liver, purified pachytene spermatocytes (PS-cytes), round spermatids (RS-tids), NIH3T3 and GC-4spc cells with antibodies to SP1, GATA1, Ccna1 and Brdt. Actin was used as loading control. Both pachytene spermatocytes and round spermatids express an actin variant, undetectable by this antibody. (TIF) [file pone.0047862.s002.tif]

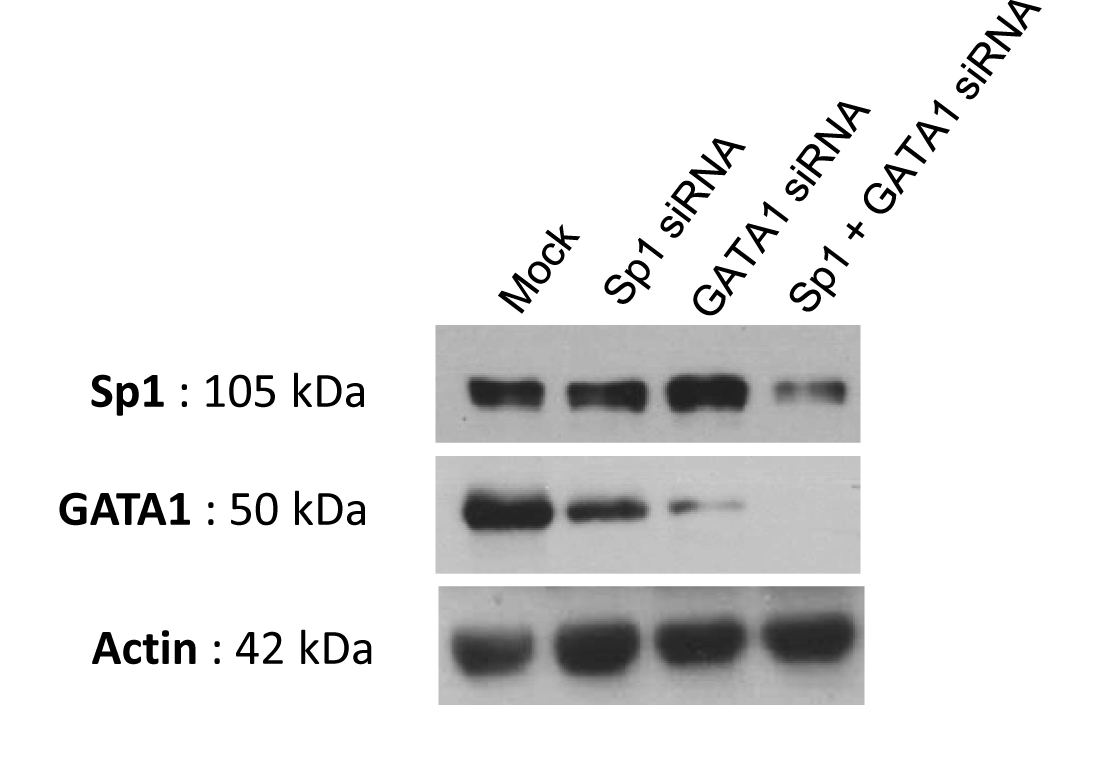

Supplement: Figure S3 — Downregulation of Sp1 and GATA1 expression. Immunoblot analysis verified the downregulation of Sp1 and GATA1 expression upon siRNA transfection. (TIF) [file pone.0047862.s003.tif]
